# Egocentric and Allocentric Reference Frames Can Flexibly Support Contextual Cueing

**DOI:** 10.3389/fpsyg.2021.711890

**Published:** 2021-08-03

**Authors:** Lei Zheng, Jan-Gabriel Dobroschke, Stefan Pollmann

**Affiliations:** ^1^Department of Experimental Psychology, Otto-von-Guericke-University, Magdeburg, Germany; ^2^Center for Behavioral Brain Sciences, Otto-von-Guericke-University, Magdeburg, Germany; ^3^Beijing Key Laboratory of Learning and Cognition and School of Psychology, Capital Normal University, Beijing, China

**Keywords:** egocentric, allocentric, reference frame, contextual cueing, visual attention

## Abstract

We investigated if contextual cueing can be guided by egocentric and allocentric reference frames. Combinations of search configurations and external frame orientations were learned during a training phase. In Experiment 1, either the frame orientation or the configuration was rotated, thereby disrupting either the allocentric or egocentric and allocentric predictions of the target location. Contextual cueing survived both of these manipulations, suggesting that it can overcome interference from both reference frames. In contrast, when changed orientations of the external frame became valid predictors of the target location in Experiment 2, we observed contextual cueing as long as one reference frame was predictive of the target location, but contextual cueing was eliminated when both reference frames were invalid. Thus, search guidance in repeated contexts can be supported by both egocentric and allocentric reference frames as long as they contain valid information about the search goal.

## Introduction

Objects are often spatially arranged in certain regularities. The human visual system has the ability to extract the regularities from sensory input in an incidental way and use them to guide visual attention. In the lab, scientists typically investigate human search behavior by asking participants to find a predefined target item among competing distractor items. Chun and Jiang (1998) designed an experiment that presented a target T among a set of distractors Ls. Importantly, and unbeknownst to their participants, in half of the trials, displays (“Repeated Display”) presented in the first block were repeated in subsequent blocks, maintaining a consistent target-distractor configuration. In the other half of the trials, newly generated displays were presented (“New Display”). Within a few blocks, the reaction time (RT) for repeated displays became significantly lower than for new displays. This search advantage, guided by invariant spatial target-distractor configurations, is indicative of contextual cueing (for reviews, Chun, 2000; Goujon et al., 2015; Jiang and Sisk, 2019; Sisk et al., 2019).

Here, we put forward that spatial reference frames (RFs) modulate contextual cueing. RFs can affect where visual attention is allocated based on spatial memory when observers encounter a familiar scene (Shelton and McNamara, 2002). One common framework divides RFs into egocentric and allocentric RFs (Klatzky, 1998; Miniaci and De Leonibus, 2018). The egocentric RF presents visual objects with respect to the observer's particular perspective, including but not limited to retinotopic, head-centered, and trunk-centered RFs. The allocentric RF, independent of the observer's perspective, presents visual objects in relation to environmental features. In a nutshell, we can distinguish the two RFs by describing their reference. For example, defining the location of an object to myself (the food shop is in front of me) uses the egocentric RF, whereas describing the shop's location relative to a landmark (the shop is to the left of a park) uses the allocentric RF.

What kind of reference frame does contextual cueing depend on? Previous work on contextual cueing has found that preserving the absolute locations of distractors in the display, but destroying the relative relations between distractors (by combining distractors from two repeated displays) suffices for contextual cueing to occur (Jiang and Wagner, 2004; Zheng and Pollmann, 2019). This could be seen as an example of an egocentric reference frame. Likewise, contextual cueing was observed when the absolute distractor locations were changed by rescaling or rotation, but their relative spatial locations were preserved (Jiang and Wagner, 2004; Zheng and Pollmann, 2019). Moreover, it has been argued that contextual cuing can be based on pairwise associations between the locations of the target and the distractors near the target (Brady and Chun, 2007). This could be argued to be an instance of an allocentric reference frame guiding contextual cueing.

**Figure 1:**
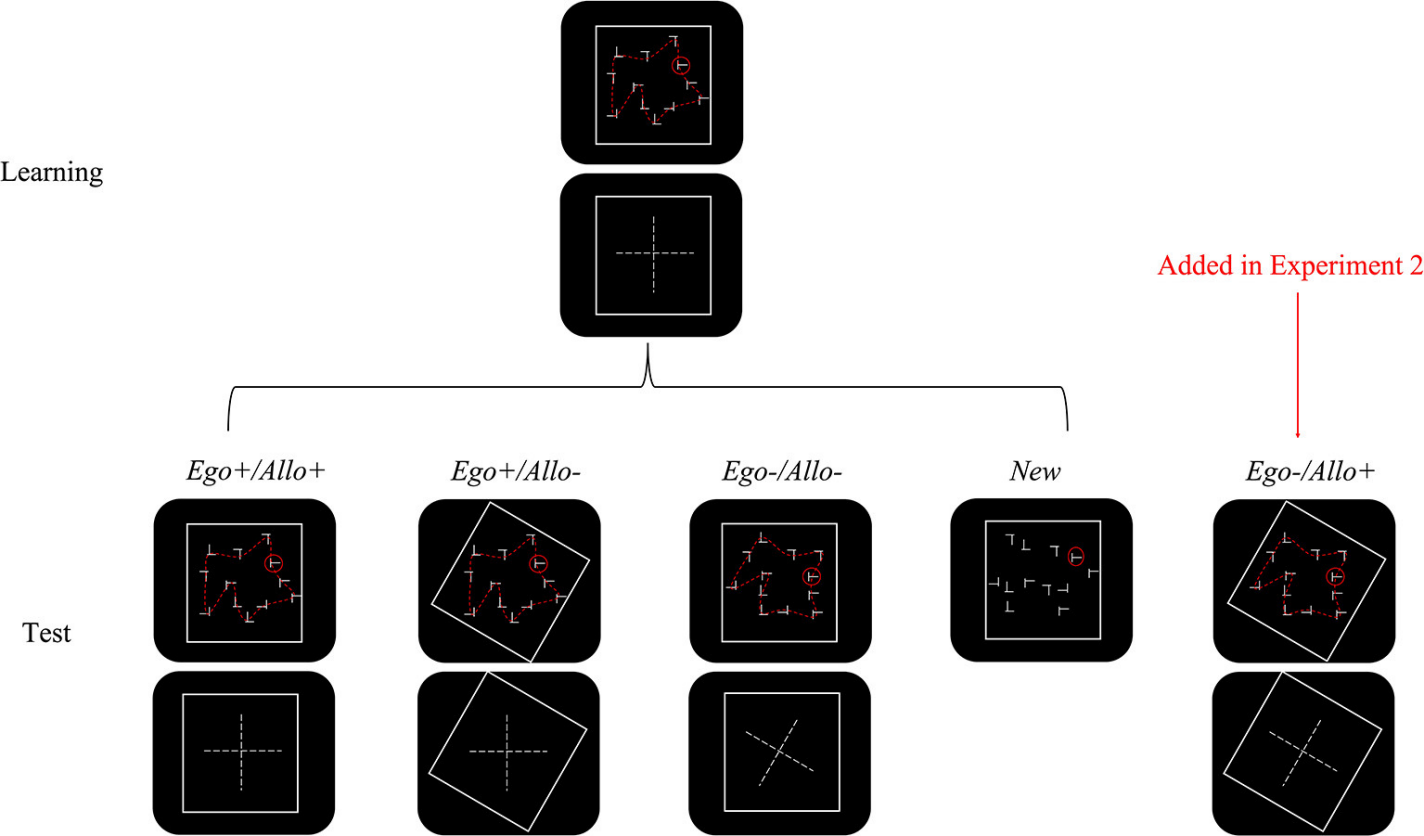
Schematic drawing of the configurations in Experiment 1 and 2. A schematic drawing panel briefly describes the displays (the cross in the middle represents the search display's configuration relative to the learning phase). Configurations in the new condition were newly generated, so there is no schematic drawing for them. The rightmost column represents one condition newly added in Experiment 2. In the actual experiment, all dash lines and the schematic drawings were not visible. For details see the section Methods.

While these examples concern the relations within items of a search display, in the present experiments, we added an external reference frame in order to investigate if contextual cueing will be modulated by it. In addition, we investigated if the reference frame underlying contextual cueing is top-down modulable. In Experiment 1, participants were repeatedly exposed to search displays within an external frame through the learning phase, enabling target-distractor configuration learning of the display but also learning to associate the display with the external frame of a certain orientation. In the subsequent test phase, by rotating the outer frame around an unchanged repeated display, we invalidated the allocentric reference frame but kept the egocentric RF intact (Figure 1). In contrast, by rotating the repeated display but keeping the external frame unchanged, we invalidated both the egocentric and allocentric reference frames. In Experiment 1a, rotation of the search display led to unequal target location probabilities, which might have confounded the measurement of contextual cueing. Therefore, Experiment 1b used displays with equal target location probability to investigate the potential contribution of target location probability cueing to Experiment 1a.

In Experiment 1, the contribution of the reference frames to contextual cueing was investigated by means of interference caused by changed—thereby invalid—reference frames. In contrast, in Experiment 2, we asked if a rotated allocentric reference frame may support search guidance when it was a valid predictor of the target location in an equally rotated display.

## Experiment 1a

### Methods

#### Participants

Twenty-six young adults (15 females and 11 males; mean age 24.12 ± 3.55 years) participated in Experiment 1a. Two participants' data were lost because of unexpected program crashes. Participants remained naive to the purpose of the research during the experiments. They had self-reported normal or corrected-to-normal vision. After the experiment, they received an €8 payment or a 1-h study credit. Participants provided written informed consent before testing. All experiments of this study were approved by the Ethics Board of the Medical Faculty of the Otto-von-Guericke University, Magdeburg.

#### Stimuli

The experiment was conducted on a screen (resolution:1,920 × 1,080 pixels; refresh rate: 120 Hz). A black cardboard with a round opening in the middle (radius = 27.2 cm) covered the screen's frame, to remove it as a potential reference frame. The stimuli were created and presented with PsychoPy3 (v3.0.0b11). Participants viewed the screen from a fixed distance of 57 cm by using a chin rest.

Each search display comprised 11 L distractor letters and one target letter T (0.4° **× ** 0.4°). The distractors had four possible directions: 0°, 90°, 180°, and 270°, while the target was rotated by 90° or 270°. The distractor *L*-shapes had an offset (of ~17%) to make them more similar to the target T (Jiang and Chun, 2001). The items were arranged on imaginary circles around central fixation with a 1.5°, 3.5°, 5.5°, and 7.2° radius with eight possible locations for each eccentricity. The eccentricities of items were balanced across each display, and the eccentricities of the target were balanced across the four configurations. Participants were asked to discriminate the target's orientation and press a corresponding key on a standard keyboard.

#### Design

After 12 practice trials that were not further analyzed, the experiment included a learning phase and a test phase. In the learning phase, participants searched 12 repeated displays for 20 blocks. These repeated displays were generated individually for each participant and presented in random sequence in each block.

The test phase consisted of four blocks. Each block contained 12 displays of each of four experimental conditions (see Figure 1), Displays were presented in random order. The four conditions varied with respect to the validity of the egocentric and allocentric cues, as follows:

*Ego*+*/Allo*+: Unchanged repeated displays from the learning phase. The plus signs indicate that both egocentric and allocentric reference frames were valid predictors of the target location.
*Ego*+*/Allo–*: Unchanged search display from the learning phase with rotated outer frame. The plus sign after *Ego* indicates that the egocentric reference frame was a valid predictor of the target location, while the minus sign after *Allo* indicates that the allocentric reference frame was an invalid predictor of the target location.
*Ego–/Allo–*: The outer frames did not rotate along with the rotated search displays. The minus signs indicate that both egocentric and allocentric reference frames were invalid predictors of the target location.
*New*: newly generated displays that shared the same set of target locations with the *Ego*+*/Allo*+ condition.

## Results

### Learning Phase

Although reaction times were the primary variable of interest, we first analyzed error rates and outlier rates (reaction times longer than three standard deviations above average and lower than 300 ms) to investigate potential speed-accuracy trade-offs. To increase statistical power, we aggregated every four consecutive blocks into a single epoch, resulting in five epochs overall. The alpha level was set at 0.05. If the main effect became significant, we ran additional *post-hoc* tests, the least significant differences (LSD), to compared data between each epoch. The mean error rate was 5.47 ± 2.44%. The one-way ANOVA showed that the error rate did not differ between epochs [*F*_(4, 119)_ = 1.069, *p* = 0.375]. The outliers rate was 3.23 ± 2.49%, with a significant main effect of epoch [*F*_(4, 119)_ = 4.160, *p* = 0.03]. *Post-hoc* LSD test showed that the outlier rate in the first epoch was significantly higher than outlier rates in the other four epochs (all *ps* < 0.003).

Error and outlier trials were excluded from the reaction times analysis. For reaction times, again, four blocks were aggregated into one epoch and the analysis procedure was identical to that for error rates and outlier rates. The one-way ANOVA yielded a significant main effect of epoch [*F*_(4, 119)_ = 4.439, *p* = 0.02] due to reduced reaction times over epochs (RT_epoch1_ = 2899 ms, RT_epoch5_ = 2531 ms). *Post-hoc* LSD showed that the reaction times in the epoch1 was significantly higher than in the other four epochs (all *ps* < 0.05, except for *p* = 0.107 between epoch1 and epoch2).

### Test Phase

One-way ANOVAs with configuration (*Ego*+*/Allo*+*, Ego*+*/Allo–, Ego–/Allo–, New*) were applied to error rates and outlier rates in test phase to rule out speed-accuracy trade-offs. The mean error rate was 2.86 ± 0.37%, and the outlier rate was 2.63 ± 0.48%. The one-way ANOVA revealed both measures did not differ between configurations [*F*_(3, 92)_ = 0.97, *p* = 0.41, and *F*_(3, 92)_ = 0.49, *p* = 0.69].

Error and outlier trials were excluded from the reaction time analysis. The repeated-measures ANOVA with configurations (*Ego*+*/Allo*+*, Ego*+*/Allo–, Ego–/Allo–, New*), and blocks (1–4) as factors was performed to investigate mean reaction times. The significant main effect of configuration [*F*_(3, 69)_ = 9.5, *p* < 0.001, *n*${}_{p}^{2}$ = 0.29] indicated reaction time differences between the four configurations, which will be further analyzed below. The significant main effect of block [*F*_(3, 69)_ = 3.10, *p* = 0.032, *n*${}_{p}^{2}$ = 0.12] reflected general learning in the test phase. The interaction between configuration and block was not significant [*F*_(9, 207)_ = 0.73, *p* = 0.678].

**Figure 2:**
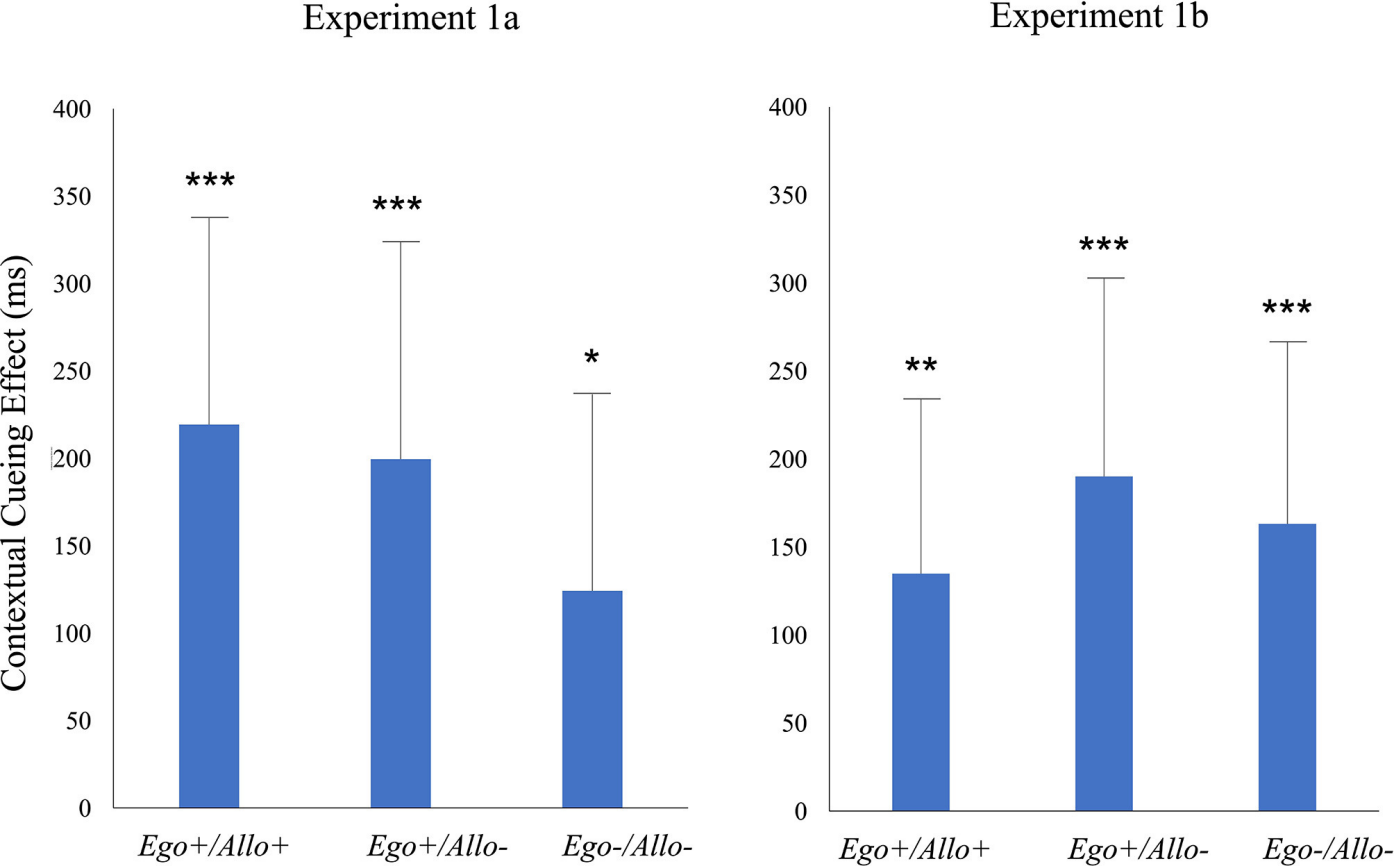
Contextual cueing in the test phase of Experiment 1a and Experiment 1b for three repeated configurations (*Ego*+*/Allo*+*; Ego*+*/Allo–; Ego–/Allo–*). Significant differences revealed by pairwise comparisons of RT_New_-RT_Repeated_ vs. zero are indicated by asterisks. Error bars represent ± 1SE of the mean. The statistical results are listed in Table 1.

To analyze the contextual cueing effect separately for the three repeated configurations (*Ego*+*/Allo*+*; Ego*+*/Allo–; Ego-/Allo-*), we respectively tested the difference between new and repeated displays (RT_New_-RT_Repeated_) vs. zero by one-tailed paired samples *T*-tests. As shown in Table 1, contextual cueing effects were significant in all three repeated configuration conditions (see Figure 2).

**Table 1:** Comparisons between RT_New_- RT_Repeated_ vs. zero in each configuration by one-tailed paired samples *T*-test.

	**Configuration**	***CCE(ms)***	***df***	***t***	***P***	***d***
EXP 1a	*Ego+/Allo+*	219	23	4.462	<0.001	1.23
	*Ego+/Allo–*	199	23	4.262	<0.001	1.29
	*Ego–/Allo–*	124	23	2.403	0.025	0.69
EXP 1b	*Ego+/Allo+*	134	24	3.331	0.003	0.95
	*Ego+/Allo–*	190	24	4.135	<0.001	1.17
	*Ego–/Allo–*	163	24	3.867	<0.001	1.10
EXP 2	*Ego+/Allo+*	150	28	4.384	<0.001	1.15
	*Ego+/Allo–*	109	28	2.346	0.013	0.61
	*Ego–/Allo–*	12	28	0.226	0.416	0.05
	*Ego–/Allo+*	112	28	1.701	0.017	0.50

Reaction times were compared by two-tailed paired samples *T*-tests to investigate significant RT-differences. The *T*-tests showed no significant difference between the three repeated configuration conditions (Table 2). Furthermore, Bayesian paired samples *T*-tests were calculated for not significant contrasts to investigate equality of RTs between conditions. These tests yielded moderate evidence for the equality of *Ego*+/*Allo*+ and *Ego*+*/Allo–*, i.e., the conditions that differed only in the validity of the allocentric cue, whereas they were close to 1 (equal probability of H_0_ and H_1_) for *Ego*+*/Allo*+ and *Ego–/Allo–*, i.e., fully valid vs. invalid cues, and for *Ego*+*/Allo–* and *Ego–/Allo–*, i.e., conditions differing in the validity of the egocentric cue (Table 2).

**Table 2:** Reaction times comparisons (two-tailed paired samples *T*-test and Bayesian paired samples *T*-test) between configurations in the test phases of Experiment 1a, Experiment 1b, and Experiment 2.

	**Configurations**		***df***	***t***	***p***	***D***	**BF_**01**_**
EXP 1a	*Ego+/Allo+*	*Ego+/Allo–*	23	0.528	0.603	0.03	4.104
		*Ego–/Allo–*	23	2.015	0.056	0.06	0.837
	*Ego+/Allo–*	*Ego-/Allo–*	23	1.923	0.067	0.26	0.966
EXP 1b	*Ego+/Allo+*	*Ego+/Allo–*	24	–1.505	0.145	0.14	1.753
		*Ego–/Allo–*	24	–0.675	0.506	0.66	3.855
	*Ego+/Allo–*	*Ego–/Allo–*	24	–0.621	0.54	0.07	3.978
*EXP 2*	*Ego+/Allo+*	*Ego+/Allo–*	28	1.168	0.253	0.11	2.733
		*Ego–/Allo+*	28	1.061	0.431	0.16	3.781
	*Ego+/Allo –*	*Ego–/Allo+*	28	–0.206	0.968	0.04	5.062
	*Ego–/Allo–*	*Ego–/Allo+*	28	1.537	0.051	0.22	4.948

## Experiment 1b

**Figure 3:**
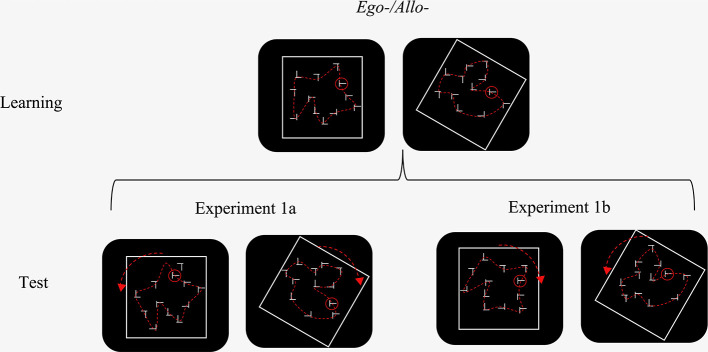
The difference between Experiment 1a and Experiment 1b in the “*Ego–/Allo–*” condition. In Experiment 1b, half of the target positions from the learning phase coincided with the other half of the target positions after rotation to remove the confound of unequal target location probabilities. In the actual experiment, the circle marking the target and the imaginary layout were not visible.

In the “*Ego–/Allo–*” configuration of Experiment 1a, the targets' absolute positions changed with the configuration's rotation, moving to a location where the target had not appeared during learning. Thus, this condition may have been more difficult than the other conditions in which target location probability may have contributed to learning. To address this issue, in Experiment 1b, we employed the same design as in Experiment 1a but with equal target location probability between locations (Geng and Behrmann, 2005; Kabata and Matsumoto, 2012; Jiang et al., 2013). Specifically, six target locations were drawn from the three outer imaginary concentric circles in the learning phase so that the polar angle between the two target positions on each circle was 30°. To keep the target location probabilities equal in the test phase, the rotated search displays were generated by 30° clockwise or counterclockwise rotation of one half each of the displays from the learning phase, see Figure 3 as an example.

### Methods

#### Participants

Twenty-five young adults (16 females and nine males; mean age 20.3 ± 2.00 years) participated in Experiment 1b. All the participants had self-reported normal or corrected-to-normal vision. None of them had been tested in Experiment 1a. After they finished the experiment, they received a €7 payment or a 1-h study credit.

#### Stimuli

The apparatus and stimuli were identical to Experiment 1a.

#### Design

We employed the same design as in Experiment 1a, except that, in learning phase, 12 target locations were drawn from the three outer imaginary concentric circles and six of them coincided with the other half of the six target positions after rotation in the test phase, keeping target location probability constant (see Figure 3).

## Results

### Learning Phase

Again, four blocks were aggregated into one epoch. The mean error rate was 5.00 ± 3.34%. The one-way ANOVA for the error rate showed a significant main effect of epoch [*F*_(4, 120)_ = 5.393, *p* < 0.001]. The outlier rate was 3.67 ± 3.47%. The one-way ANOVA on outliers showed a significant main effect of epoch [*F*_(4, 120)_ = 2.247, *p* < 0.001]. The *post-hoc* LSD tests showed that the outlier rates and error rates in epochs 1 and 2 were significantly higher than outlier rates and error rates in epochs 4 and 5 (all *ps* < 0.05, except for *p* = 0.058 for comparisons between error rates in epoch 2 and epoch 4).

Error and outlier trials were excluded from the reaction time analysis. The one-way ANOVA on reaction times yielded a significant main effect of epoch [*F*_(4, 120)_ = 3.652, *p* = 0.008] due to the reduced reaction times over epochs (RT_epoch1_ = 2,873 ms, RT_epoch5_ = 2,391 ms). The *post-hoc* LSD showed that the reaction time in epoch 1 was significantly higher than in epochs 4 and 5 (*ps* < 0.05) but not significantly higher than epoch 2 (*p* = 0.333) and epoch 3 (*p* = 0.082).

### Test Phase

The mean error rate was 3.37 ± 0.24%. The one-way ANOVA revealed that it did not differ between configurations [*F*_(3, 99)_ = 0.931, *p* = 0.43]. The outlier rate was 4.06 ± 1.49%. The significant main effect of configuration [*F*_(3, 99)_ = 3.577, *p* = 0.02] indicated different outlier rates between the configurations. The *post-hoc* LSD showed the outlier rate in the New configuration was significantly higher than the three repeated configurations (all *ps* < 0.05). Error and outlier trials were excluded from the subsequent analysis for reaction times.

Again, a repeated-measures ANOVA with configurations (*Ego*+*/Allo*+*, Ego*+*/Allo–, Ego–/Allo–, New*) and blocks (1–4) as factors was performed to investigate mean reaction times. The significant main effect of configuration [*F*_(3, 72)_ = 8.132, *p* < 0.001, *n*${}_{p}^{2}$ = 0.253] indicated reaction time differences between the four configurations, which will be further analyzed below. The significant main effect of block [*F*_(3, 72)_ = 4.486, *p* = 0.006, *n*${}_{p}^{2}$ = 0.157] reflected general learning. The interaction between configuration and block was not significant [*F*_(9, 216)_ = 0.825, *p* = 0.593].

The RT_New_-RT_Repeated_ differences in the three repeated configurations (*Ego*+*/Allo*+; *Ego*+*/Allo–; Ego–/Allo–*) were significantly higher than zero (Table 1), indicating significant contextual cueing in the three repeated configuration conditions (Figure 2).

We compared the potential difference of reaction times between the three configurations (*Ego*+*/Allo*+; *Ego*+*/Allo–; Ego–/Allo–*) with two-tailed paired samples *T*-tests. There were no significant differences. To further test for equality of reaction times between configurations, we calculated Bayesian paired samples *T*-tests. These tests yielded moderate evidence for the equality of *Ego*+*/Allo*+ and *Ego–/Allo–*, differing in the validity of both cue types, of *Ego*+*/Allo–* and *Ego–/Allo–*, differing in the validity of the egocentric cue, and anecdotal evidence for the equality of *Ego*+*/Allo*+ and *Ego*+*/Allo–*, differing in the validity of the allocentric cue (Table 2).

### Interim Discussion

#### The Contribution of Target Location Probability to Search Performance

Although the amount of contextual cueing effect in *Ego–/Allo–* configuration did not differ significantly between Experiment 1a and Experiment 1b (independent samples *T*-test: *t*_(47)_ = 0.59, *p* = 0.56), we observed a numerically stronger contextual cueing effect in Experiment 1b (163 ms) compared to Experiment 1a (124 ms). We inferred that the weaker contextual cueing effect in Experiment 1a might have been due to the target appearing in low-probability locations after rotation. However, the weaker but still significant contextual cueing effect in the *Ego–/Allo–* condition of Experiment 1a indicated that a potential target location probability cueing effect was not sufficient to eliminate the contextual cueing effect.

#### Summary of the Results of Experiment 1

Across Experiment 1a and Experiment 1b, we found that disrupting the allocentric and egocentric reference frames could not eliminate the search advantage for repeated displays. Specifically, we found that contextual cueing is preserved when a rotated external frame suggests that the search display is likewise rotated, but it is not (*Ego*+*/Allo–*). We even found contextual cueing when the learned display was rotated within the unchanged external frame, so that both egocentric and allocentric reference frames were changed (*Ego–/Allo–*). At first sight, this may suggest that both reference frames are irrelevant for contextual cueing. However, in the test phase of Experiment 1, rotating only the configuration (*Ego–/Allo–*) and rotating only the frame (*Ego*+*/Allo–*) led to an imbalance of the validity of the egocentric and allocentric reference frames, in that the egocentric reference frame was valid in 50% of trials (two out of four conditions: *Ego*+*/Allo*+ and *Ego*+*/Allo–*) whereas the allocentric reference frame was valid only in 25% of trials (the *Ego*+*/Allo*+ condition). This may have led participants (incidentally) to ignore the frame orientation during the test phase. Instead, they may have used the display orientation itself as the allocentric reference, which would explain the intact contextual cueing effect in the *Ego–/Allo–* condition.

## Experiment 2

The design of Experiment 1 was based on an interference logic—would invalid allocentric and egocentric reference frames eliminate contextual cueing? In contrast, Experiment 2 included a new condition in which the external frame and the display were rotated by the same angle, so that the changed allocentric RF was a valid predictor of the target location. This design change also removed a potential weakness of Experiment 1, namely the unequal validity between the egocentric and allocentric reference frames.

### Methods

#### Participants

Thirty young adults (18 females and 12 males; mean age 21.46 ± 3.21 years) participated in Experiment 2. One participant's data were excluded because the error rate (16.88%) was higher than three deviations above the average (3.90%). None of them had participated in Experiment 1. All the participants had self-reported normal or corrected-to-normal vision. All of them provided written, informed consent to take part in the experiment. After they finished the task, they received an €8 payment or a 1-h study credit.

#### Stimuli

The apparatus, stimuli, and trial sequence were identical to those in Experiment 1b, except that the items' (T and Ls) were rotated along with the configuration, forming orientations of 30°, 120°, 210°, and 300°, in addition to the non-rotated orientations of 0°, 90°, 180°, and 270°.

### Design

The design was identical to those in Experiment 1b, except that the *Ego–/Allo*+ condition was newly added and the number of trials per condition was reduced to eight per block. In the *Ego–/Allo*+ condition, the outer frames rotated along with the rotated search displays, making the changed allocentric reference frame a valid predictor of the target location (see Figure 1).

## Results

### Learning Phase

The mean error rate was 3.32 ± 3.03%, and the mean outlier rate was 3.38 ± 4.36%. The one-way ANOVA yielded a significant main effect for both measures [error rate: *F*_(4, 144)_ = 2.528, *p* = 0.043; outlier rate: *F*_(4, 44)_ = 3.373, *p* = 0.011]. The *post-hoc* LSD tests showed that the error rate in the fifth epoch was significantly lower than error rates in the previous four epochs (all *ps* < 0.05), and the outlier rate in the first epoch was significantly higher than error rates in the other four epochs (all *ps* < 0.01).

Error and outlier trials were excluded from the reaction time analysis. The one-way ANOVA on reaction times yielded a significant main effect of epoch [*F*_(4, 144)_ = 15.729, *p* < 0.001] due to the reduced reaction times over epochs (RT_epoch1_ = 2,725 ms, RT_epoch5_ = 1,970 ms). LSD showed that the reaction time in epoch 1 was significantly higher than in the other four epochs (all *ps* < 0.05).

### Test Phase

The mean error rate was 1.68 ± 0.76% and the mean outlier rate was 2.56 ± 0.84%. Both measures did not differ between configurations [*F*_(4, 144)_ = 1.428, *p* = 0.228, and *F*_(4, 144)_ = 2.325, *p* = 0.06, respectively].

The repeated-measures ANOVA with configurations (*Ego*+*/Allo*+*, Ego*+*/Allo–, Ego–/Allo–, Ego–/Allo*+*, New*) and blocks (1–4) as factors was performed to investigate mean reaction times. The significant main effect of configuration [*F*_(3, 72)_ = 8.132, *p* < 0.001, *n*${}_{p}^{2}$ = 0.253] indicated reaction times differences between the configurations, which will be further analyzed below. The significant main effect of block [*F*_(3, 72)_ = 4.486, *p* = 0.006, *n*${}_{p}^{2}$ = 0.157] reflected general learning. The interaction between configuration and block was not significant [*F*_(9, 216)_ = 0.825, *p* = 0.593].

**Figure 4:**
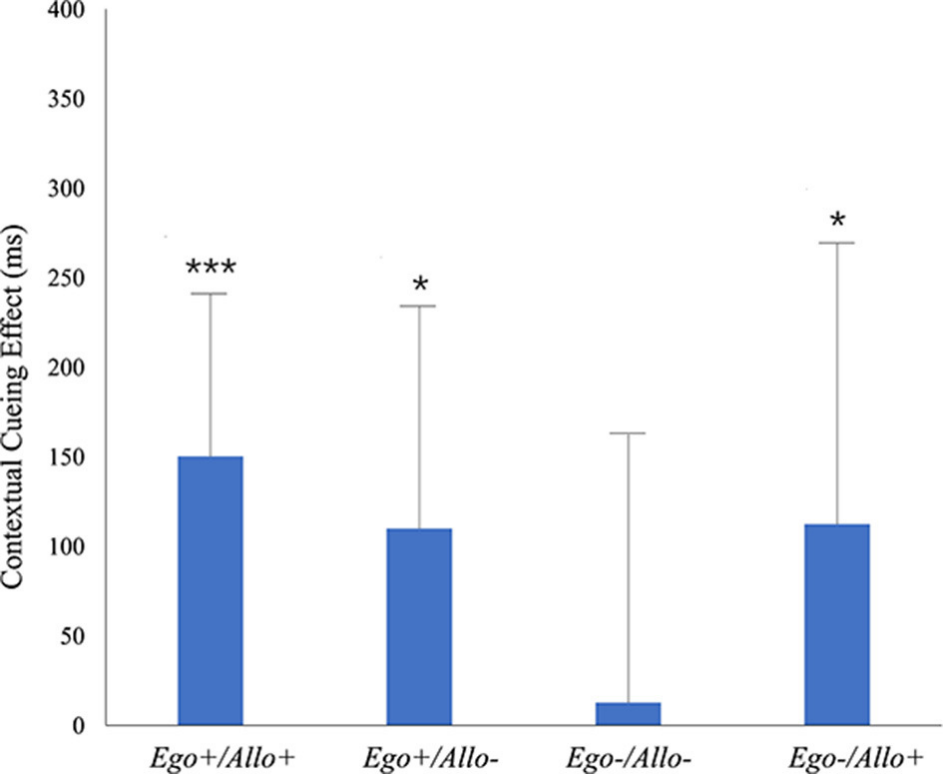
Contextual cueing in the test phase of Experiment 2 for four repeated configurations (*Ego*+*/Allo*+*, Ego*+*/Allo–, Ego–/Allo–, Ego–/Allo*+). Significant differences revealed by pairwise comparisons of RT_New_-RT_Repeated_ and zero are indicated by black asterisks. Error bars represent SE of the mean. The statistical results can be seen in Table 1.

To analyze the contextual cueing effect separately for the four repeated configurations (*Ego*+*/Allo*+; *Ego*+*/Allo–; Ego–/Allo–; Ego–/Allo*+) yielded contextual cueing, we respectively compared the RT_New_–RT_Repeated_ vs. zero contrast for the four repeated conditions. As the results of the one-tailed paired samples *T*-test (shown in Table 1), contextual cueing effects in the *Ego*+*/Allo*+, *Ego*+*/Allo–*, and *Ego–/Allo*+ configurations were significant, but no significant contextual cueing was observed in the *Ego–/Allo–* configuration, i.e., the condition with both invalid egocentric and allocentric cues (see Figure 4).

We compared the reaction times between the four repeated conditions (*Ego*+*/Allo*+; *Ego*+*/Allo–; Ego–/Allo–; Ego–/Allo*+) by two-tailed paired samples *T*-tests (Table 2). Whereas the results indicated no significant difference between *Ego*+*/Allo*+, *Ego*+*/Allo–*, and *Ego–/Allo*+, the search advantage for *Ego–/Allo–* was significantly lower than for *Ego*+*/Allo*+ and *Ego*+*/Allo–*, and marginally lower than for *Ego–/Allo*+. Bayesian paired samples *T*-tests again investigated the equality of RTs in non-significant contrasts. Moderate evidence was yielded for the equalities of *Ego*+*/Allo*+ and *Ego–/Allo*+, conditions differing in the egocentric cue validity, *Ego–/Allo–* and of *Ego–/Allo*+, differing in the validity of the allocentric cue, and of *Ego*+*/Allo– and Ego–/Allo*+, conditions with one valid and one invalid cue type. Anecdotal evidence for RT-equality was observed for the *Ego*+*/Allo*+ and *Ego*+*/Allo–* conditions, with valid egocentric cue, but differing in the validity of the allocentric cue.

## Interim Discussion

Again, we observed contextual cueing if at least one of the reference frames was preserved from training to test. However, in contrast to Experiment 1, no contextual cueing was observed when both reference frames yielded invalid predictions (*Ego–/Allo–*). This was expected if participants relied more on the external reference frame (and less on allocentric information from the search display itself) because of the frame's higher overall validity (Zang et al., 2018).

## General Discussion

The present study provides an initial exploration of the dependence of contextual cueing on egocentric and allocentric reference frames. In Experiment 1, we invalidated either the validity of an external reference frame alone or together with an additional invalidation of the egocentric reference frame. Both manipulations did not lead to significant reductions in the size of contextual cueing, compared with fully repeated display / frame combinations.

Experiment 1a might have been affected by a potential confound between contextual cueing and target location probability cueing (Geng and Behrmann, 2005; Kabata and Matsumoto, 2012; Jiang et al., 2013, also see review Sisk et al., 2019), leading to potentially lower contextual cueing scores when the display was rotated (*Ego–/Allo–* condition, marginal difference between *Ego–/Allo–* and *Ego*+*/Allo*+ conditions). However, when this confound was removed in Experiment 1b, no significant difference between the contextual cueing scores of *Ego–/Allo–* and *Ego*+*/Allo*+ was observed, indicating that contextual cueing was obtained in spite of both reference frames being invalid predictors of the target location.

Experiment 1 investigated the contribution of egocentric and allocentric reference frames with an interference logic. We investigated if contextual cueing was reduced when either the external frame or the display itself was rotated, relative to fully repeated displays. However, this procedure may have led participants to ignore the external frame's orientation and focus on the display itself. This view is supported by similar results in previous experiments where only the search displays were repeated without an external frame and contextual cueing was observed in spite of display rotation (Jiang and Wagner, 2004; Zheng and Pollmann, 2019).

In Experiment 2, we introduced trials with a rotated external frame that validly predicted the target location, because we wanted to know if changes of the allocentric reference frame that were valid predictors of the target location could be used for contextual cueing. We observed comparable contextual cueing scores when either the allocentric or egocentric reference frame was valid, but the other was not, suggesting that both egocentric and allocentric reference frames could support contextual cueing. Of note, if only one reference frame was valid (*Ego*+*/Allo–* and *Ego–/Allo*+), numerically lower contextual cueing effects were observed than for fully repeated display (40 and 37 ms, respectively). It might be tempting to speculate if both RFs contribute jointly to contextual cueing. However, besides that these differences were not significant, to test this further, one would need to systematically vary the magnitude of rotation of the display and the external frame in order to see if this affects the size of the contextual cueing effect (Chua and Chun, 2003).

Strikingly, contextual cueing was abolished when both reference frames were invalid in Experiment 2 (*Ego–/Allo–*). Not surprisingly, the Bayesian paired samples *T*-tests provided moderate or anecdotal evidence for longer reaction times in the *Ego–/Allo–* condition than the other three repeated conditions (Rouder et al., 2009). This contrasted with a significant contextual cueing effect in the *Ego–/Allo–* condition in Experiment 1, which was of equal size with the *Ego*+*/Allo*+ and *Ego*+*/Allo–* conditions. As mentioned, in Experiment 1, the allocentric reference frame had a low predictiveness of the target location and therefore might not have been used to retrieve memory traces of repeated displays. Apparently, the increased validity of the external reference frame prompted its use for search guidance in repeated displays (Zang et al., 2018). Note, this difference cannot be due to learning because the learning phases of Experiments 1 and 2 did not differ in validity. The different validities of the reference frames occurred only in the test phase. Therefore, the presence vs. absence of contextual cueing when both reference frames were invalid must have been due to memory retrieval or search guidance processes rather than due to configuration learning. This emphasizes the importance to distinguish between learning and expression of learning in contextual cueing (Frensch et al., 1998; Manginelli et al., 2013a,b).

The term “egocentric” or “allocentric” has assumed a very general meaning (Klatzky, 1998; Wang, 2017) and was used here to manifest the relationship between items and observers and between items and landmarks (Andersen and Enriquez, 2006). Further research might specify the contribution of retinotopic or head-centered reference frames (Jiang and Swallow, 2013). In related work, the importance of a body-centered reference frame has been demonstrated in spatial priming (Ball et al., 2010) and of an anatomical reference frame in tactile contextual cuing (Assumpção et al., 2018). Future studies might also investigate three-dimensional reference frames (Issartel et al., 2016). Chua and Chun (2003) trained participants on a virtual 3-D display viewed from a single viewpoint but tested at various rotations away from the training viewpoint. The results showed search advantages for trained repeated displays decreased as the rotation angular from training viewpoint to testing viewpoint increased. It might be worthwhile to investigate if a valid external 3-dimensional reference frame could prevent this view-point dependent reduction of contextual cueing.

It might be argued that in contextual cueing, the display itself contains allocentric information, i.e., the spatial relations between search items (Ball et al., 2009). As mentioned, this may have contributed to the intact contextual cueing effect in the *Ego–/Allo–* condition of Experiment 1. However, we believe that we have shown in Experiment 2 that an external reference frame can influence contextual cueing independent of the spatial information that can be gained from the display itself. One difference between Experiment 1 and Experiment 2 was that in the latter, the search items orientations were rotated along with the whole display, which may have supported the coding of the relative spatial positions of display items, i.e., allocentric information. However, this seems not to have affected contextual cueing in any major way because these items' rotations occurred in the *Ego–/Allo–* and the *Ego–/Allo*+ conditions, where we observed no contextual cueing in the former, but in the latter. Thus, the difference in contextual cueing between these conditions can only be due to the external frame validity.

It may also seem puzzling that the *Ego*+*/Allo–* condition led to solid contextual cueing in Experiment 1 and Experiment 2 alike. One could argue that contextual cueing should be reduced in Experiment 2 because the allocentric cue is overall more valid than in Experiment 1, so that if it is invalid, as in the *Ego*+*/Allo–* condition, it should interfere with efficient search guidance. Obviously, this was not the case, suggesting that either valid egocentric or allocentric cues alone are sufficient to guide search in repeated displays. Note that the size of contextual cueing was numerically smaller and more variable in the *Ego*+*/Allo–* and *Ego–/Allo*+ conditions than in the fully repeated (*Ego*+*/Allo*+) displays. While these differences failed to be significant, they also failed to yield robust evidence for equal size of contextual cueing in the Bayes tests, particularly between the *Ego*+ *Allo*+ and *Ego*+ *Allo–* conditions. Thus, there is tentative evidence for an interfering effect of misleading egocentric and allocentric cues. However, it remains a worthwhile question for future research why the interference of misleading cues is smaller than the benefit of valid cues.

Egocentric and allocentric processes have also been discussed in the larger domain of spatial memory. Some research indicates that the two reference frames act simultaneously in spatial memory (e.g., McNamara, 2002; Mou et al., 2004; Waller and Hodgson, 2006; also see a review Avraamides and Kelly, 2008). In other cases, knowledge for one reference frame is developed in the relative absence of the other (Wang and Spelke, 2000; Ishikawa and Montello, 2006; Jiang and Swallow, 2013; Jiang et al., 2013). Our study contributes to this discussion in two ways: First, it provides evidence that the reference frames in spatial memory can be top-down modulated, implying the adoption of reference frames influenced by intent of the participants. That gives a possible explanation why Jiang et al. (2013) found that participants showed probability cueing, another form of incidental spatial learning, with the egocentric reference frame, but with the allocentric reference frame when they were explicitly told about the regularity of search displays. Second, our findings highlight the need for consideration of cue validity when investigating reference frames in spatial memory.

## Conclusions

We investigated the contribution of egocentric and allocentric reference frames to contextual cueing. Learned combinations of display configurations and orientations of an external frame could be used flexibly, depending on their probability to predict the target location.

## Data Availability Statement

The data are available at https://osf.io/ypbem/.

## Ethics Statement

The studies involving human participants were reviewed and approved by Ethical Board of the Medical Faculty of the Otto-von-Guericke University, Magdeburg. The patients/participants provided their written informed consent to participate in this study.

## Author Contributions

LZ and SP: experimental design, interpretation of data, and manuscript writing. LZ and J-GD: data collection. All authors contributed to the article and approved the submitted version.

## Conflict of Interest

The authors declare that the research was conducted in the absence of any commercial or financial relationships that could be construed as a potential conflict of interest.

## Publisher's Note

All claims expressed in this article are solely those of the authors and do not necessarily represent those of their affiliated organizations, or those of the publisher, the editors and the reviewers. Any product that may be evaluated in this article, or claim that may be made by its manufacturer, is not guaranteed or endorsed by the publisher.
